# Development of HTLV-1 associated myelopathy/tropical spastic paraparesis in a patient with simian T-lymphotropic virus type 1-like infection.

**DOI:** 10.1186/1742-4690-12-S1-O30

**Published:** 2015-08-28

**Authors:** Yoshimi Enose-Akahata, Breanna Caruso, Benjamin Haner, Raya Massoud, Bridgette Jeanne Billioux, Joan Ohayon, William M Switzer, Steven Jacobson

**Affiliations:** 1 Viral Immunology Section, Neuroimmunology Branch, National Institute of Neurological Disorders and Stroke, National Institutes of Health, Bethesda, MD, USA; 2 Laboratory Branch, Division of HIV/AIDS, National Center for HIV, Hepatitis, STD, and TB Prevention, Centers for Disease Control and Prevention, Atlanta, GA 30329, USA

## 

Virus transmission from various wild and domestic animals contributes to increased risk of emerging infectious diseases in human populations. HTLV-1 is a human retrovirus associated with acute T-cell leukemia (ATL) and HTLV-1-associated myelopathy/tropical spastic paraparesis (HAM/TSP), which originated from zoonotic transmission from various African and Asian nonhuman primates (NHPs). Similar to HTLV-1, the simian counterpart, STLV-1, causes chronic infection and leukemia and lymphoma in naturally infected monkeys. However, other clinical syndromes typically seen in human such as a chronic progressive myelopathy have not been observed in NHPs. Little is also known about the development of any neurologic and inflammatory diseases in human populations infected with STLV-1-like viruses following NHP exposure. We identified and analyzed the complete genome of a primate T lymphotropic virus type 1 (PTLV-1) isolated from a patient with typical HAM/TSP who resides in the United States but was born in Liberia. Using a novel droplet digital PCR for the detection of the HTLV-1 tax gene, the proviral load in PBMC was 14.01%; however there was a distinct difference in fluorescence amplitude compared to all other H!M/TSP patient's, suggesting viral heterogeneity. A complete PTLV-1 proviral genome was amplified from DNA extracted from the PBMCs of the HAM/TSP patient using PCR to generate nine overlapping subgenomic fragments. Phylogenetic analysis of PTLV-1 env and LTR regions showed the virus was highly related with PTLV-1 from sooty mangabey monkeys and humans exposed from NHPs in West Africa. These results suggest the patient is likely infected with STLV-1, suggesting for the first time that viral transmission from monkey to human may be associated with a chronic progressive neurologic disease.

